# Reduced cerebello-cerebral functional connectivity correlates with disease severity and impaired white matter integrity in Friedreich ataxia

**DOI:** 10.1007/s00415-023-11637-x

**Published:** 2023-03-01

**Authors:** Rebecca Kerestes, Hannah Cummins, Nellie Georgiou-Karistianis, Louisa P. Selvadurai, Louise A. Corben, Martin B. Delatycki, Gary F. Egan, Ian H. Harding

**Affiliations:** 1 Department of Neuroscience, Central Clinical School, Monash University, Melbourne, Australia; 2 Turner Institute for Brain and Mental Health, School of Psychological Sciences, Monash University, Melbourne, Australia; 3 Bruce Lefroy Centre for Genetic Health Research, Murdoch Children’s Research Institute, Melbourne, Australia; 4 Monash Biomedical Imaging, Monash University, Melbourne, VIC 3800 Australia

**Keywords:** Friedreich ataxia, Cerebellum, Resting state, Anterior, Superior, Dentate

## Abstract

Friedreich ataxia (FRDA) is a rare, inherited neurodegenerative disease characterised in most cases by progressive and debilitating motor dysfunction. Degeneration of cerebellar white matter pathways have been previously reported, alongside indications of cerebello-cerebral functional alterations. In this work, we examine resting-state functional connectivity changes within cerebello-cerebral circuits, and their associations with disease severity (Scale for the Assessment and Rating of Ataxia [SARA]), psychomotor function (speeded and paced finger tapping), and white matter integrity (diffusion tensor imaging) in 35 adults with FRDA and 45 age and sex-matched controls. Voxel-wise seed-based functional connectivity was assessed for three cerebellar cortical regions (anterior lobe, lobules I-V; superior posterior lobe, lobules VI-VIIB; inferior posterior lobe, lobules VIIIA-IX) and two dentate nucleus seeds (dorsal and ventral). Compared to controls, people with FRDA showed significantly reduced connectivity between the anterior cerebellum and bilateral pre/postcentral gyri, and between the superior posterior cerebellum and left dorsolateral PFC. Greater disease severity correlated with lower connectivity in these circuits. Lower anterior cerebellum-motor cortex functional connectivity also correlated with slower speeded finger tapping and less fractional anisotropy in the superior cerebellar peduncles, internal capsule, and precentral white matter in the FRDA cohort. There were no significant between-group differences in inferior posterior cerebellar or dentate nucleus connectivity. This study indicates that altered cerebello-cerebral functional connectivity is associated with functional status and white matter damage in cerebellar efferent pathways in people with FRDA, particularly in motor circuits.

**Supplementary Information:**

The online version contains supplementary material available at 10.1007/s00415-023-11637-x.

## Introduction

Friedreich ataxia (FRDA) is a recessively inherited neurodegenerative disorder principally characterised by progressive motor incoordination [1] that manifests secondary to degeneration in the spinal cord, cerebellum, and cerebello-cerebral connections [2]. Quantitative structural magnetic resonance imaging (MRI) studies have been key to defining the pattern and evolution of anatomical brain changes in people with FRDA. MRI studies indicate early, robust, and progressive volume loss in the dentate nuclei [3, 4], which are the primary sites of pathology in the brain [5]. Atrophy of the cerebellar cortex is more subtle and considered a late feature of the disease. This volume loss is particularly weighted to the anterior lobe and adjacent areas of the posterior lobe (i.e., lobules I-VI) [2, 6]], which connect to motor and premotor areas of the cerebral cortex. Loss of volume and axonal integrity within the superior cerebellar peduncles (SCP), which contain the ascending efferent fibres of the cerebellum is also a consistent and robust finding [2, 7–15]. These anatomical changes result in a progressive decline in cerebellar innervation of the cerebral cortex, particularly impacting (pre)motor circuits.

The consequences of disruption in the cerebello-cerebral pathways are observed in functional MRI studies of individuals with FRDA, as detailed in a comprehensive review by Vavla and colleagues [16]. Motor and cognitive tasks elicit abnormal functional activations in the cerebellum and cerebrum, relative to healthy controls [16]. This typically involves reduced activation in task-relevant cerebellar and cerebral regions, although in some cases, increased cerebral activation—suggested to reflect compensatory activity—has also been observed [3, 17–20]. More limited investigations of cerebello-cerebral functional connectivity have also been undertaken in FRDA. During performance of cognitive tasks, reduced cerebello-cerebral connectivity has been observed between task-relevant brain regions [3, 6], while resting-state functional connectivity—estimated based on the spontaneous fluctuation of neuroimaging signals in the absence of a specific task—is also reduced between frontal areas implicated in executive processes and the contralateral cerebellar cortex [21]. However, a recent magnetoencephalography (MEG) study also reported increased resting cerebello-cerebral connectivity specifically in people with first symptom onset in adolescence or adulthood (relative to childhood) [22, 23]. These studies provide clear evidence of functional disruptions in cerebello-cerebral circuits relevant to particular task contexts in people with FRDA.

In this study, we extend on this work by providing a more targeted assessment of connectivity changes between different anatomical and/or functional zones of the cerebellum and the cerebral cortex. Using resting-state fMRI, we first quantify differences between people with FRDA and healthy controls in the functional connectivity of three distinct anatomical subregions of the cerebellar cortex: (i) the anterior lobe, which is predominantly associated with motor processes and has reciprocal connections with cerebral motor and premotor cortices [24]; (ii) the superior posterior lobe, which is primarily associated with non-motor processes [25] and has reciprocal connections with the prefrontal cortex, posterior parietal, and superior temporal cerebral cortices [26, 27]; and (iii) the inferior posterior lobe which is connected with cerebral motor and somatosensory cortices [24, 28]. We also map the functional connectivity of the dorsal (motor) and ventral (non-motor) zones of the dentate nuclei [29, 30]. We then investigate relationships between functional connectivity, disease severity, psychomotor function, and white matter integrity assessed by diffusion tensor imaging (DTI) in the FRDA cohort.

## Methods

A total of 35 people with FRDA, homozygous for a GAA expansion in intron one of *FXN* (mean age 35.39 ± 13.35), and 45 age and sex-matched healthy controls (mean age 36.05 ± 13.12) were included in the final data analyses (Table 1). Participants were recruited from two Melbourne-based studies: (i) IMAGE-FRDA (*n* = 30 controls, 24 FRDA) and (ii) INFLAM-FRDA (*n* = 15 controls, 11 FRDA) as previously described [2, 3, 31, 32]. We hereafter refer to these sites as Site 1 and Site 2, respectively.

**Table 1:** Demographics, disease metrics and psychomotor characteristics by group

	FRDA (*n* = 35)	HC (*n* = 45)	*S*tatistic	*p*
	*M* (SD) or *N* (%)	*M* (SD) or *N* (%)		
Sex (F)	15 (42.86)	20 (44.44)	*χ*^*2*^ = 0.02	0.89
Age	35.39 (13.35)	36.05 (13.12)	*t* = 0.22	0.83
Age at onset	18.20 (8.07)	–	–	–
Disease duration (years)	17.22 (10.24)	–	–	–
SARA	20.82 (7.41)	–	–	–
Paced Tapping Consistency (1/ms)	0.013 (0.006)	0.025 (0.007)	*t* = 8.44	< 0.001
Speeded Tapping (ms)	518.00 (222.74)	211.70 (24.88)	*t* = − 9.17	< 0.001
FA_SCP	0.43 (0.05)	0.55 (0.03)	*F* = 244.70	< 0.001
FA_MCP	0.48 (0.05)	0.51 (0.05)	*F* = 19.52	< 0.001
FA_CST	0.51 (0.05)	0.55 (0.04)	*F* = 67.71	< 0.001

*SARA* Scale for the Assessment and Rating of Ataxia, *ms* milliseconds, *FA_SCP* fractional anisotropy of the superior cerebellar peduncles, *FA_MCP* fractional anisotropy of the middle cerebellar peduncles, *FA_CST* fractional anisotropy of the corticospinal tract. Site included as covariate in ANCOVAs comparing fractional anisotropy outcomes for each group

Seven additional participants with FRDA (3 from Site 1, 4 from Site 2) were excluded due to excessive motion during scanning (average framewise displacement > 0.5 mm), and one control was excluded due to an fMRI acquisition that did not include the whole cerebellum. In cases where participants were enrolled in both studies, only data from one was included. Participants with FRDA had no comorbid neurological or psychiatric diagnoses. Severity of ataxia impairment was quantified using the Scale for the Assessment and Rating of Ataxia (SARA; [33]), scored from 0 (no ataxia) to 40 (most severe ataxia). This study was sanctioned by either the Monash Health or Monash University Human Research Ethics Committees (13201B and 2017–7810 respectively). All participants provided written informed consent.

### Magnetic resonance imaging

Magnetic resonance images were collected using a 3-Tesla Siemens Skyra scanner for Site 1 and a 3 T Siemens PET-MR Biograph scanner for Site 2. High-resolution T1-weighted images were acquired as follows. Site 1: magnetization prepared rapid gradient-echo (MPRAGE), TR = 1900 ms, TE = 2.19 ms, TI = 900 ms, flip angle = 9°, FOV = 256 × 256 mm, 176 sagittal slices, 1 mm isotropic voxels. Site 2: MP2RAGE, TR = 5000 ms, TE = 3.43 ms, TI1 = 700 ms, TI2 = 2500 ms, flip angles = 4°,5°, FOV = 256 × 256 mm, 192 sagittal slices, 1 mm isotropic voxels.

Resting-state blood oxygen-level dependent (BOLD) echo-planar fMRI was acquired as follows. Site 1: TR = 2500 ms, TE = 30 ms, axial slices = 44, voxel size = 3 mm isotropic, 200 volumes (8.5 min), phase acceleration (GRAPPA) = 2. Site 2: TR = 1390 ms, TE = 30 ms, axial slices = 48, voxel size = 3 mm isotropic, 320 volumes (7.75 min), phase acceleration (CAIPIRINHA) = 2, slice acceleration = 2. In both cases, participants were instructed to keep their eyes open and focus on a fixation cross roughly in the centre of their visual field.

Diffusion-weighted MRI was acquired as follows. Site 1: TR = 11600 ms, TE = 108 ms, axial slices = 60, voxel size = 2.1 mm isotropic, 69 volumes (5 × b0, 64 × b3000), phase acceleration (GRAPPA) = 2. Site 2: TR = 4800 ms, TE = 104 ms, axial slices = 58, voxel size = 2.5 mm isotropic, 74 volumes (5 × b0, 3 × b200, 5 × b500, 30 × b1000, 30 × b2500), slice acceleration = 2.

### Cerebellum parcellation & seed definition

Cerebellar lobules were automatically segmented using Automatic Cerebellum Anatomical Parcellation using U-Net with Locally Constrained Optimization (ACAPULCO; version 0.2.1; [34]; https://gitlab.com/shuohan/acapulco), and quality checked in accordance with the ENIGMA cerebellum parcellation protocol [35]; https://enigma.ini.usc.edu/protocols/imaging-protocols/). Three bilateral cerebellar cortical regions-of-interest (ROI) were defined for each participant: (i) anterior lobe (lobules I-V), (ii) superior posterior lobe (lobules VI-VII) and (iii) inferior posterior lobe (lobules VIII-IX). Two dentate nucleus ROIs were additionally defined using 2 mm radial spheres centred on MNI coordinates defined by previous literature [29, 36]: (i) dorsal seed: *x* = 12, *y* = − 57, *z* = − 30 and (ii) ventral seed: *x* = 17, *y* =  − 65, *z* =  − 35. The dentate seeds were defined in the right hemisphere only.

### fMRI preprocessing

The fMRI data were pre-processed and voxel-wise seed-based functional connectivity were derived using the CONN functional connectivity toolbox (version 20b; https://www.nitrc.org/projects/conn) [37]. Preprocessing included spatial realignment and unwarping, temporal slice-timing correction, coregistration of the functional to the structural MRI data, normalisation to MNI space based on segmentation and nonlinear warping of the structural image, and spatial smoothing (8 mm FWHM Gaussian kernel). Volumes with framewise displacement > 0.5 mm or global BOLD signal changes > 5 standard deviations were flagged for each participant. The following confounds were regressed out of the fMRI timeseries: each outlier volume, 12 estimated subject-motion parameters (3 translation and 3 rotation parameters and their first-order derivatives), and average signals extracted from white matter and cerebrospinal fluid masks. The data were further denoised using a bandpass filter of 0.008–0.09 Hz.

### fMRI statistical analysis

For each participant, seed-to-voxel connectivity maps were generated for each of the 5 seed ROIs. For each seed, between-group differences, controlling for site and age, were inferred using non-parametric cluster permutation tests (SnPM toolbox; 10,000 permutations; cluster-forming threshold *T* = 2.5). For the dentate nucleus seeds, both seeds were included in the same regression model (i.e., dorsal seed connectivity while controlling for ventral seed connectivity, and vice-versa) as previously described [29, 38]. Inference was restricted to relevant grey matter cerebral regions defined by the average whole-brain positive functional connectivity of that seed across all participants (*p* < 0.001). Final results were thresholded at *p* < 0.05_FWE_ cluster-level corrected.

### Diffusion MRI preprocessing and statistical analysis

The diffusion-weighted MRI was processed using the FSL TBSS pipeline to derive fractional anisotropy values for each participant, mapped to a template white matter skeleton (https://fsl.fmrib.ox.ac.uk/fsl/fslwiki/TBSS) [39]. For each region showing a between-group cerebello-cerebral functional connectivity difference, linear associations between functional connectivity and fractional anisotropy were assessed in the FRDA group, controlling for site. Voxel-wise analyses across the full white matter skeleton were undertaken using non-parametric permutation (5000 permutations) and threshold-free cluster enhancement, implemented in FSL randomise (https://fsl.fmrib.ox.ac.uk/fsl/fslwiki/Randomise) [40]. Mean FA values for the superior cerebellar peduncle (SCP) and middle cerebellar peduncle (MCP) were also extracted using the JHU ICBM-DTI-81 atlas, and for the corticospinal tract (CST) from the JHU White Matter Tractography atlas for ROI-based analyses.

### Behavioural motor tasks

Participants performed computerised speeded and paced finger tapping tasks prior to scanning [31, 41, 42]. The speeded finger tapping task (STAP) assesses psychomotor speed and requires rapid non-dominant index finger tapping for 10 s, repeated 5 times. The outcome measure was the mean intertap interval across all trials; higher values represent worse performance. The paced tapping task (PTAP) assesses timing and coordination, requiring alternating button pressing with the left and right thumbs in time with a repeating 1.8 Hz tone. After 11 taps, the tone was discontinued and participants continued tapping at the same pace for 32 additional taps. The outcome measure was the reciprocal of the standard deviation of the intertap intervals; higher values indicate better performance.

### ROI statistical analysis

All analyses were run in JASP version 0.16 (JASP team 2022). Correlation analyses in the FRDA cohort were conducted to assess linear associations between cerebello-cerebral functional connectivity for each connection that showed a significant between-group difference and: (i) SARA score, (ii) disease duration, (iii) age of symptom onset (AO), (iv) speeded finger tapping, (v) paced finger tapping, and (vi, vii, viii) fractional anisotropy in the corticospinal tract (CST), superior cerebellar peduncle (SCP) and the middle cerebellar peduncle (MCP). Site was included as a covariate in all models.

In addition, supplementary exploratory analyses were undertaken to assess relationships between the above clinical, behavioural, and diffusion measures and functional connectivity between each cerebellum ROI and its associated cerebral target (i.e., explicit mask, defined above). That is, inference was not restricted to areas showing FRDA vs. control between-group connectivity differences. These analyses were conducted in both the FRDA and the control groups, separately. Results were Bonferroni corrected to account for exploration across the 5 cerebellar ROIs, yielding a corrected *p* threshold of 0.01 (i.e., 0.05/5).

## Results

### Cerebellar cortex functional connectivity

**Fig. 1:**
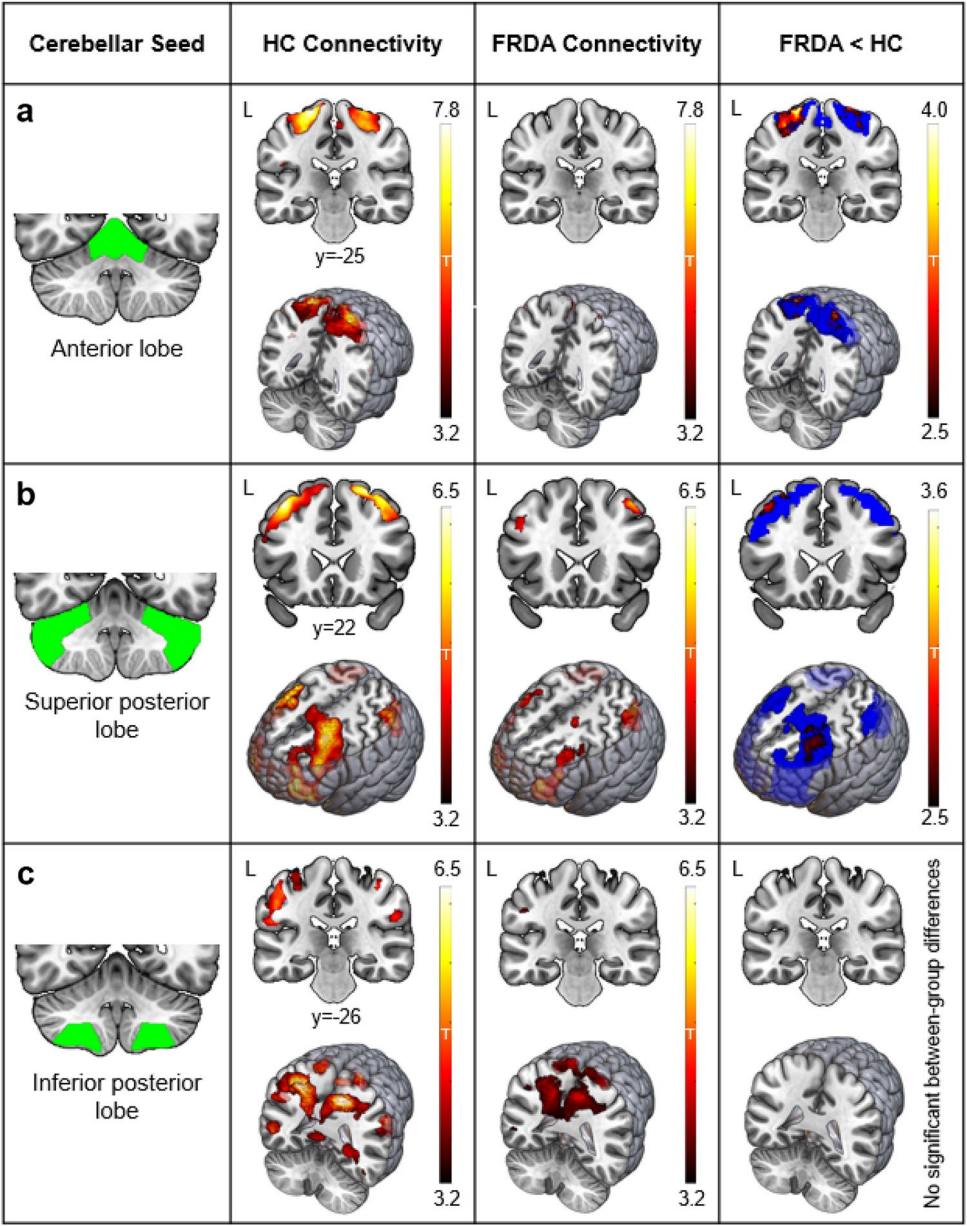
Cerebellar Cortex–Cerebral Cortex Functional Connectivity. Leftmost panel: Coronal sections displaying three cerebellar seeds of interest in green (**a**, anterior lobe; **b**, superior posterior lobe; **c**, inferior posterior lobe). Middle panels: Within-group positive connectivity in the healthy control (HC) and Friedreich ataxia (FRDA) groups (*p* < 0.001 uncorrected). Rightmost panel: Significant between-group differences (FRDA < HC) in positive functional connectivity (*p*_FWE_ < 0.05 cluster-level corrected; hot colours), within explicit masks defined by the average positive functional connectivity across all participants (blue). *Y* = MNI coordinate of the coronal slice

As illustrated in Fig. 1, the anterior lobe cerebellar seed was preferentially connected to the precentral gyrus (e.g., motor network); the superior posterior lobe to the lateral prefrontal, superior parietal, and inferior temporal cortices (i.e., executive network); and the inferior posterior lobe to the superior frontal, medial-superior parietal, and superior-lateral occipital cortices (i.e., attention network).

Individuals with FRDA, relative to controls, showed significantly reduced functional connectivity of the anterior cerebellum to bilateral precentral and postcentral gyri (Fig. 1a, left: *x* =  − 26, *y* =  − 28, *z* = 72; cluster size = 667; *p*_FWE_ = 0.002; right: *x* = 22, *y* =  − 20, *z* = 76; cluster size = 232; *p*_FWE_ = 0.023). Within-group data visualisations (Fig. 1a; uncorrected *p* < 0.001) indicated robust and widespread connectivity in controls, which is absent in the FRDA group.

The FRDA cohort, relative to controls, also showed significantly reduced functional connectivity between the superior posterior cerebellum and the left dorsolateral prefrontal cortex (Fig. 1b; *x* =  − 40, *y* = 30, *z* = 42; cluster size = 388; *p*_FWE_ = 0.017). Uncorrected effect size maps (Supplementary Fig. 1) suggest that this effect may be bilateral, but the right hemisphere did not survive cluster thresholding. Within-group visualisations (Fig. 1b) indicate robust and widespread connectivity in the controls, which is attenuated in FRDA particularly in dorsolateral prefrontal areas.

There were no significant between-group differences observed in inferior posterior cerebellar connectivity, with both groups showing a similar pattern and magnitude of connectivity within the mask (Fig. 1c). No between-group differences were evident even at uncorrected thresholds (Supplementary Fig. 1).

### Dentate nucleus functional connectivity

**Fig. 2:**
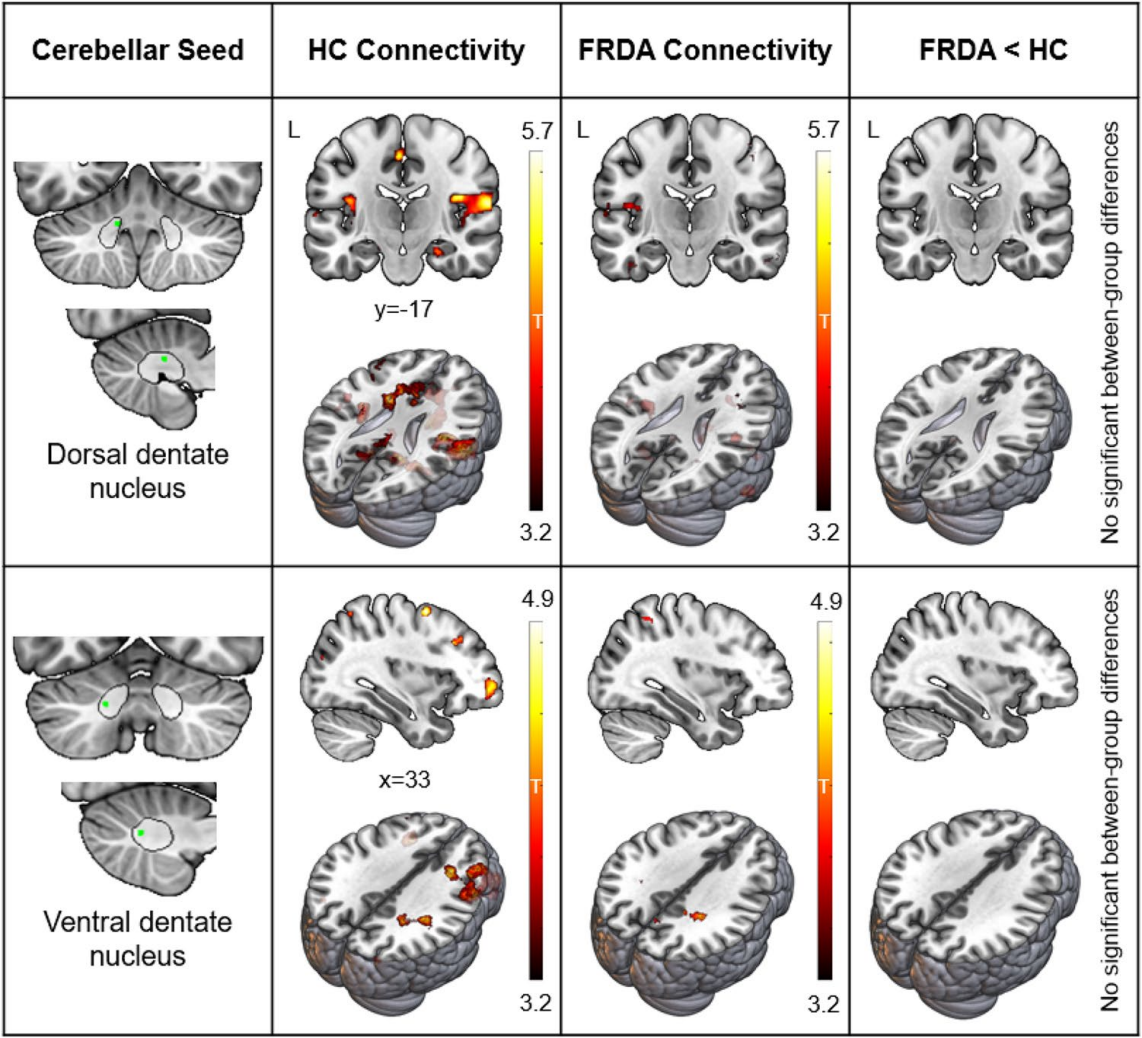
Dentate Nucleus–Cerebral Cortex Functional Connectivity. Leftmost panel: Coronal and sagittal sections displaying the dentate nucleus (outlined in black) and seed regions (green). Middle panels: Within-group positive connectivity in the healthy control (HC) and Friedreich ataxia (FRDA) groups, *p* < 0.001 uncorrected. Rightmost panel: Absence of significant between-group (i.e., FRDA < HC) connectivity differences (*p*_FWE_ < 0.05 cluster-level corrected). *X*, *y* = MNI coordinates of the sagittal and coronal slices. See Supplementary Fig. 1 for between-group differences at *p* < 0.05 uncorrected

As illustrated in Fig. 2, the dorsal dentate nucleus seed was preferentially connected to frontal operculum, anterior cingulate, and precentral gyrus. The ventral dentate nucleus seed was most strongly connected with the lateral prefrontal and superior parietal cortices.

There were no significant between-group differences in dentate nucleus functional connectivity. Uncorrected between-group (Supplementary Fig. 1) and within-group (Fig. 2) visualisations suggest a trend towards reduced connectivity in the FRDA cohort, but these effects are not robust to multiple comparison corrections.

### Clinical and behavioural correlations

In the FRDA cohort, clinical and behavioural correlations were assessed relative to functional connectivity strength in the two connections that demonstrated significant between-group differences: anterior cerebellum to primary motor cortex, and superior posterior cerebellum to left dorsolateral prefrontal cortex (DLPFC).

**Fig. 3:**
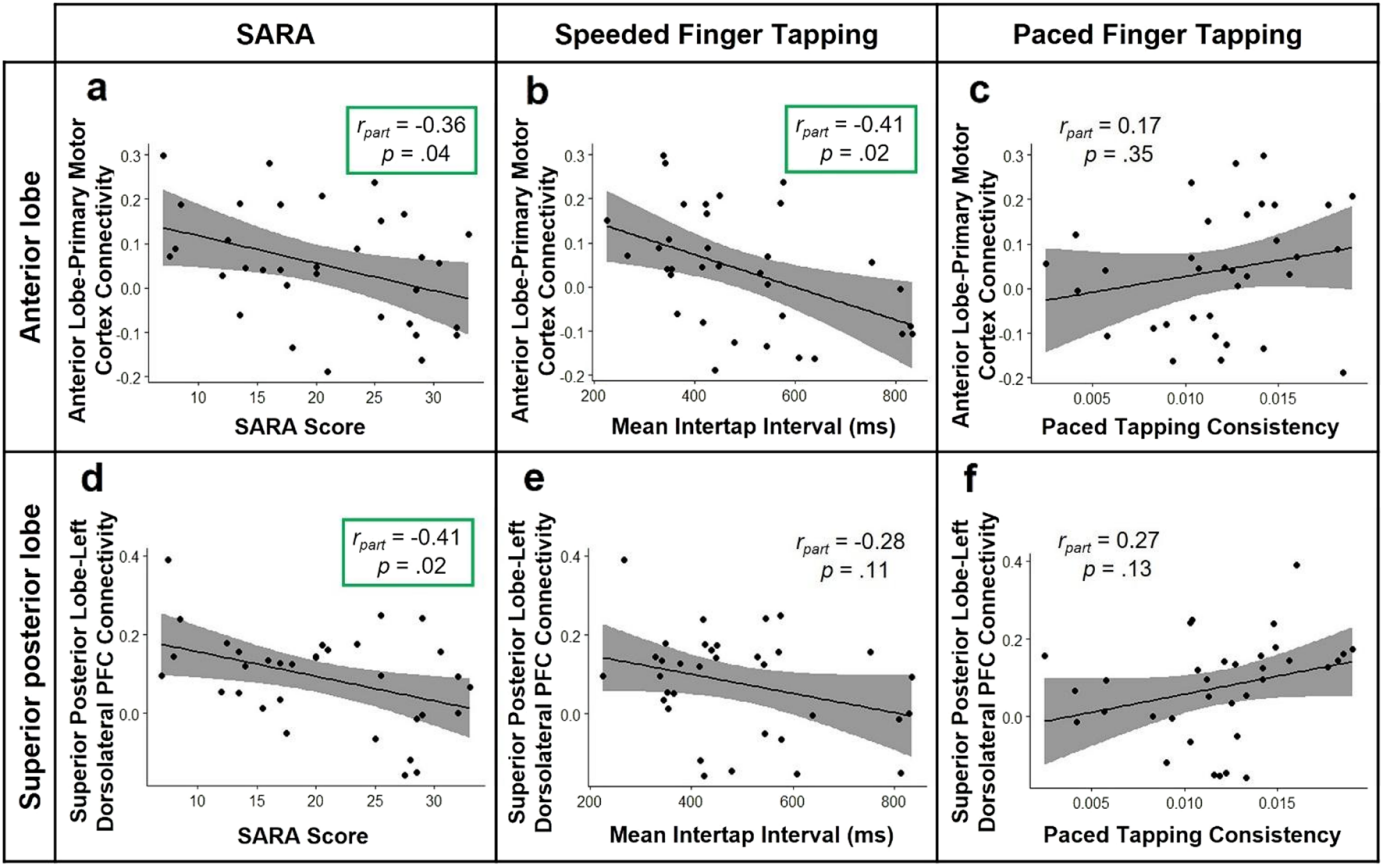
Linear relationships between cerebello-cerebral functional connectivity (in connections showing significant between-group differences) and disease severity (**a, d**) or psychomotor function (**b, c, e, f)** in the Friedreich ataxia cohort. *SARA* Scale for the Assessment and Rating of Ataxia, *ms* milliseconds, *r*_*part*_ partial correlation controlling for site

Significant negative correlations were found between the SARA score (disease severity) and both anterior cerebellum—primary motor cortex (*r* =  − 0.36, *p* = 0.04) and superior posterior—DLPFC (*r* =  − 0.41, *p* = 0.02) functional connectivity (Fig. 3). There were no significant correlations between connectivity and disease duration or symptom onset age (Supplementary Table 1).

Anterior cerebellum—primary motor connectivity negatively correlated with speeded tapping in individuals with FRDA, such that reduced functional connectivity was associated with a slower tapping rate (*r* =  − 0.41, *p* = 0.02; Fig. 3). No significant associations were observed for paced tapping task performance (Fig. 3, Supplementary Table 1).

Exploratory analyses in connections that did not show between-group differences additionally revealed a significant positive correlation between ventral dentate connectivity and paced tapping scores in individuals with FRDA (*r* = 0.53, *p* < 0.001; Supplementary Fig. 2; Supplementary Table 2). In the healthy control group, there were no significant correlations between cerebellum connectivity and either of the finger tapping tasks (Supplementary Table 3).

### White matter integrity vs. functional connectivity

**Fig. 4:**
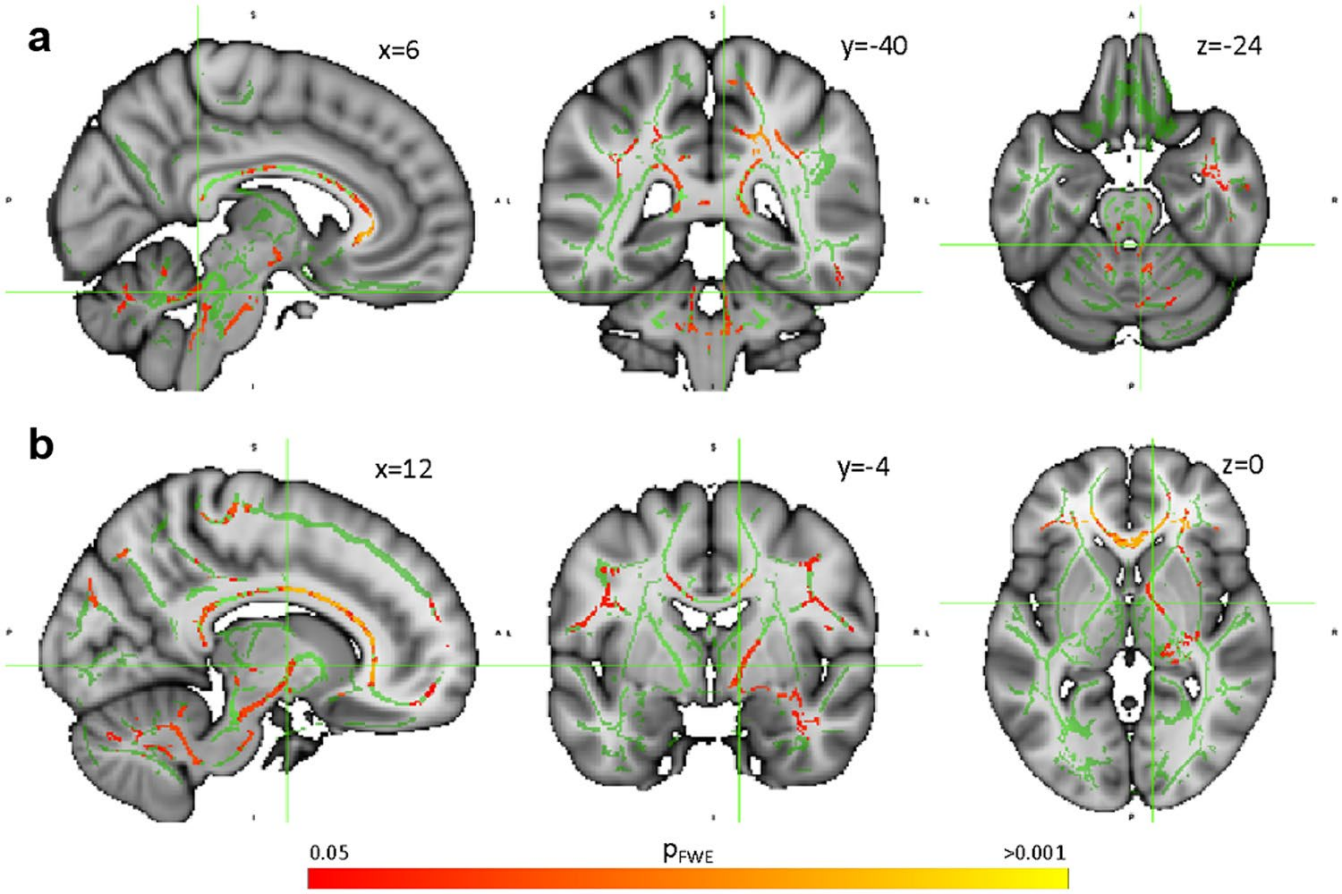
Significant positive correlations between fractional anisotropy and anterior cerebellum-primary motor cortex functional connectivity in the FRDA cohort (yellow–red; *p*_FWE_ < 0.05) projected on a skeleton of the white matter; areas of the skeleton that are not significantly correlated are depicted in green. Depictions are provided at the level of the cerebellum (**a**) and basal ganglia (**b**)

Widespread reductions in fractional anisotropy were evident in the FRDA cohort relative to controls (Supplementary Fig. 2). In the FRDA group, weaker functional connectivity between the anterior cerebellum and the primary motor cortex (i.e., the connection depicted in Fig. 1A) was associated with lower fractional anisotropy—a proxy measure of white matter integrity—in the bilateral superior cerebellar peduncles, right cerebral peduncle, right internal capsule, and adjacent to the precentral gyrus (i.e., ascending cerebello-cerebral pathways). Widespread involvement of the corpus callosum is also observed (Fig. 4).

These associations did not reach significance when considering the entire SCP, MCP, or CST as ROIs (Supplementary Table 1), suggesting relatively focal effects. There were no significant voxel-wise or ROIs correlations between superior posterior—DLPFC connectivity and fractional anisotropy in the FRDA group (Supplementary Table 1). However, ventral dentate nucleus functional connectivity with the cerebral cortex did correlate with fractional anisotropy in the SCP ROI (Supplementary Fig. 2; Table 2). There were no significant correlations between functional connectivity and fractional anisotropy in the control group (Supplementary Table 3).

## Discussion

Using a multi-modal MRI approach, we show that disrupted cerebello-cerebral functional connectivity in people with FRDA is associated with disease severity, motor functioning, and white matter integrity. Impaired functional connectivity was particularly evident between the cerebellar and cerebral cortices, rather than between the dentate nuclei and cerebrum. The convergence of abnormalities in brain function, brain structure, clinical status, and behavioural performance was most evident in the motor system, although not exclusively. This work demonstrates that the functional consequences of neurodegeneration in people with FRDA are widespread, affecting whole-brain functional circuits. These alterations are evident in a task-free context (i.e., resting state), reflecting a trait-like general property of FRDA pathophysiology.

Alterations in cerebello-cerebral functional connectivity were present in both motor (anterior lobe) and non-motor (superior posterior lobe) circuits in FRDA. These findings are consistent with prior structural MRI studies that report reduced cerebellar grey matter volume in these regions [2, 6, 43, 44], alongside atrophy in cerebral motor and premotor cortices [2, 43, 45]. Prior task-based fMRI studies also report alterations in cerebral and cerebellar functional activations in motor networks during finger tapping tasks [16, 17, 19, 31, 44, 46]. Here, we show that the magnitude of deficits in cerebello-cerebral coupling in motor networks correlates with the severity of ataxia symptoms and psychomotor performance in people with FRDA.

Although motor deficits are the most debilitating and salient features of FRDA, cognitive changes are also apparent [47]. Alongside connectivity changes in the motor system, we also observed deficits in non-motor systems involving connections between the superior posterior lobe and lateral prefrontal cortex. This finding replicates Cocozza and colleagues [21], who also found reduced resting-state functional connectivity in this pathway in people with FRDA. Task-based studies have similarly shown reduced activation in prefrontal areas, and impaired task-related connectivity between the superior posterior lobe and prefrontal cortices, during performance of cognitive tasks [3, 6, 18]. Importantly, however, connectivity between the superior posterior lobe and other areas of the cortex outside of the lateral prefrontal cortex did not appear to be greatly impacted, and inferior posterior cerebellar connectivity was spared. Aberrant communication between the cerebellum and cerebrum therefore appears to be a progressive and intrinsic property of the brain in FRDA that manifests in specific functional circuits.

Extensive prior work has shown that cerebello-cerebral white matter tracts, the efferent axonal pathways of the cerebellum, are robustly impacted in people with FRDA [2, 7–15]. Here, we show that the strength of cerebello-cerebral *functional* connectivity is directly related to the integrity of these underlying *anatomical* pathways in FRDA. This observation is in-line with established principles of structure–function coupling of brain connectivity [48, 49]. This relationship was particularly evident in the motor system and specific to ascending cerebellar efferent tracts (i.e., superior cerebellar peduncles). This observation further supports the conceptualisation of FRDA as a disease principally impacting bottom-up inputs from the cerebellum (and spinal cord) to the cerebrum, with a relative sparing of top-down signalling [2, 3, 50, 51].

Unexpectedly, evidence for reduced functional connectivity of the dentate nucleus in people with FRDA, relative to controls, did not reach statistical significance in this study. The dentate nuclei are the primary sites of neuropathology in FRDA, and the main efferent hubs of the cerebellum which give rise to the superior cerebellar peduncles [5]. Atrophy of the dentate nuclei and superior cerebellar peduncles are robust and early features of FRDA which manifest prior to anatomical changes in the cerebellar cortex [2, 52]], strongly supporting the hypothesis that dentate nucleus functional connectivity would be disrupted. However, our null findings may be influenced by methodological considerations. Firstly, as noted above, the functional networks implicated in FRDA appear to be relatively specific, and it is possible that the seed regions we specified in the dentate nucleus did not capture maximally impacted connections. Indeed, the selected dorsal seed region was principally connected to cerebral premotor and salience network regions, rather than the primary motor system. The fMRI signal measured in the dentate nucleus also has a significantly lower signal-to-noise ratio (SNR) relative to the cerebellar and cerebral cortices due to its location and tissue properties (i.e., high iron concentration) [53]. This reduced SNR decreases the likelihood of detecting significant effects at the same statistical threshold as the cortical seeds. In support, we find evidence of reduced dentato-cerebral connectivity below our established threshold for statistical detection with correction for multiple comparisons (Supplementary Fig. 2). Although this cannot be interpreted as evidence of a between-group difference, it does indicate that this study may have been underpowered to detect these effects and supports larger-scale future replication.

Limitations of this work must be considered. Herein, we tested targeted hypotheses regarding cerebello-cerebral connectivity of defined anatomical regions of the cerebellum based on established disease models. However, exploratory whole-brain investigations in larger cohorts using graph theory, independent components analysis, or other spatial unconstrained whole-brain approaches may help further define the nature of abnormal brain connectivity in people with FRDA. These approaches should be considered within large-scale imaging consortia, such as ENIGMA-Ataxia [2]. Different acquisition protocols and hardware were used for data collection at each site; however, an age and sex-matched control group was available for each site, site effects were accounted for during statistical modelling, and data processing was harmonised, minimising the impact of this methodological variability. Furthermore, heterogeneity in clinical and behavioural measures available across the two sites precluded more detailed and wide-ranging analyses of disease features (e.g., cognitive profiles). In addition, this study only included adults, and our FRDA cohort did not encompass the full range of disease severity, limiting generalisability. Finally, although our functional connectivity results and correlations with diffusion metrics largely imply abnormal connectivity of ascending tracts from the cerebellum to the cerebrum, these techniques cannot definitively disentangle the involvement of bottom-up vs top-down interactions between the cerebrum and cerebellum.

In conclusion, this study provides novel evidence of alterations in cerebello-cerebral connectivity that correlate with disease status, psychomotor function, and white matter integrity in people with FRDA. These changes are robust in primary motor systems, with more limited and spatially targeted effects in non-motor circuits. These results contribute to the understanding of FRDA as a “whole-brain” disease, and establish links between different imaging markers of systems-level abnormalities. This work motivates the need for subsequent longitudinal studies to investigate associations between cerebello-cerebral network changes and symptom progression, and their potential as biomarkers for disease tracking.

## Supplementary Information

Below is the link to the electronic supplementary material.

Supplementary file1 (PDF 678 KB)

## Data Availability

Group-level summary data is available upon reasonable request to the corresponding author; raw data is available through research collaboration.
