# Supplementary material for: Reduced cerebello-cerebral functional connectivity correlates with disease severity and impaired white matter integrity in Friedreich ataxia
Source: J Neurol. 2023 Mar 1;270(5):2360–9. doi: 10.1007/s00415-023-11637-x (PMC10130106; doi:10.1007/s00415-023-11637-x)
Supplement: Supplementary file 1 — Supplementary file1 (PDF 678 KB) [file 415_2023_11637_MOESM1_ESM.pdf]

Reduced cerebello-cerebral functional connectivity correlates with disease severity and impaired white matter integrity in Friedreich ataxia

Journal of Neurology

Rebecca Kerestes*[1], Hannah Cummins*[1], Nellie Georgiou-Karistianis[2], Louisa P. Selvadurai[1], Louise A Corben[3], Martin B Delatycki[3], Gary F Egan[2,4], and Ian H Harding[1,4]

*Equal Contribution

1 Department of Neuroscience, Central Clinical School, Monash University, Melbourne
2 Turner Institute for Brain and Mental Health, School of Psychological Sciences, Monash University, Melbourne
3 Bruce Lefroy Centre for Genetic Health Research, Murdoch Children's Research Institute, Melbourne
4 Monash Biomedical Imaging, Monash University, Melbourne

Correspondence to: Dr. Ian Harding
Department of Neuroscience, Monash University, VIC 3800
ian.harding@monash.edu

Supplementary Information

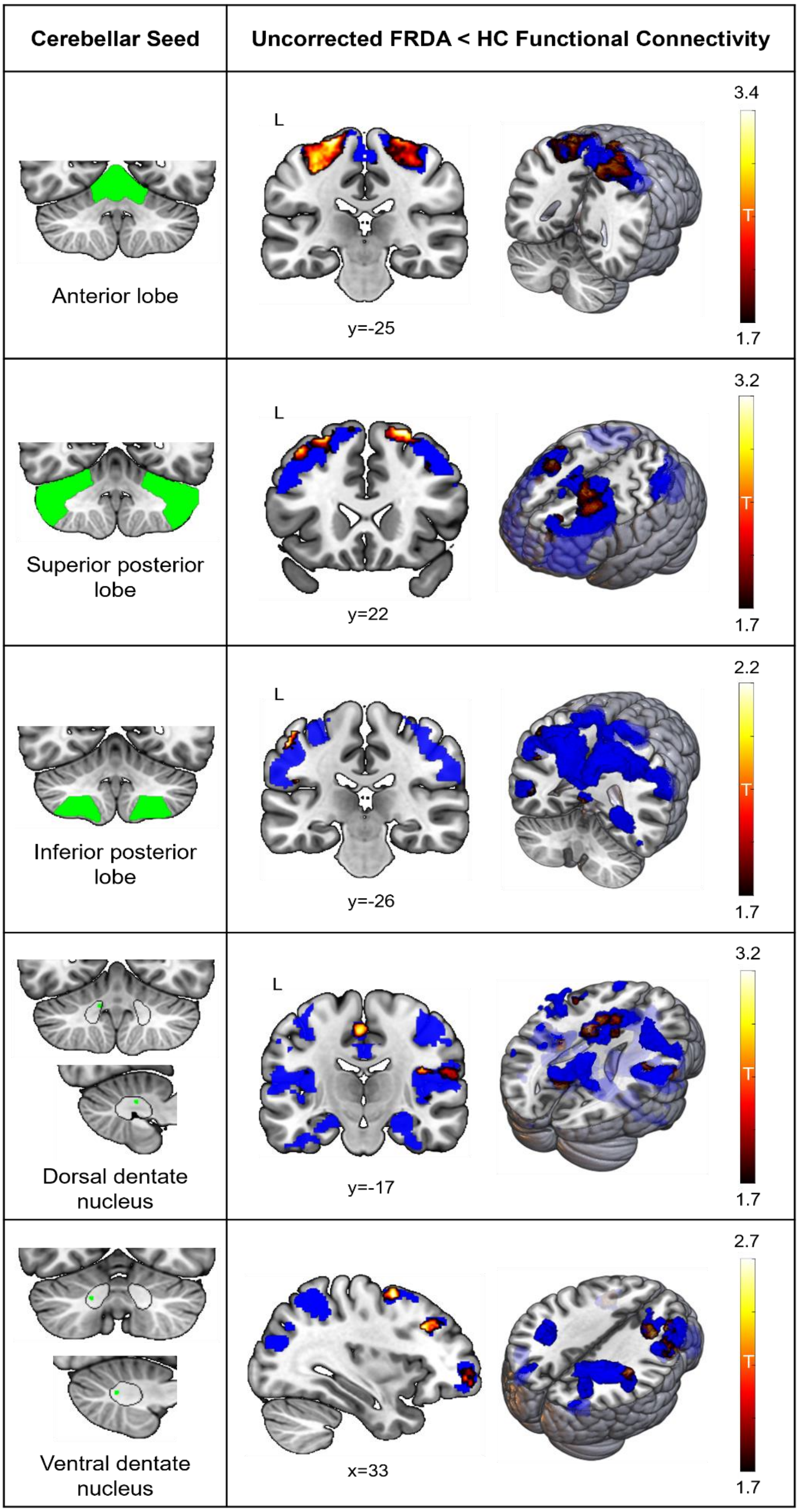

Supplementary Fig. 1 Uncorrected group differences in cerebello-cerebral functional connectivity**. Left panel:** Coronal and sagittal sections displaying the five cerebellar seeds of interest in green. For the final two seeds, the dentate nucleus is further outlined in black within the cerebellum. **Right panel:** Yellow-black colouring on coronal or sagittal sections and 3D renderings represents between-group differences (FRDA < HC) in cerebello-cerebral functional connectivity at a height threshold of $p < .05$ uncorrected for the seed shown in the left panel. Blue colouring represents the explicit masks used in restricting these inferences to relevant cerebral grey matter regions. x and y = MNI coordinates.

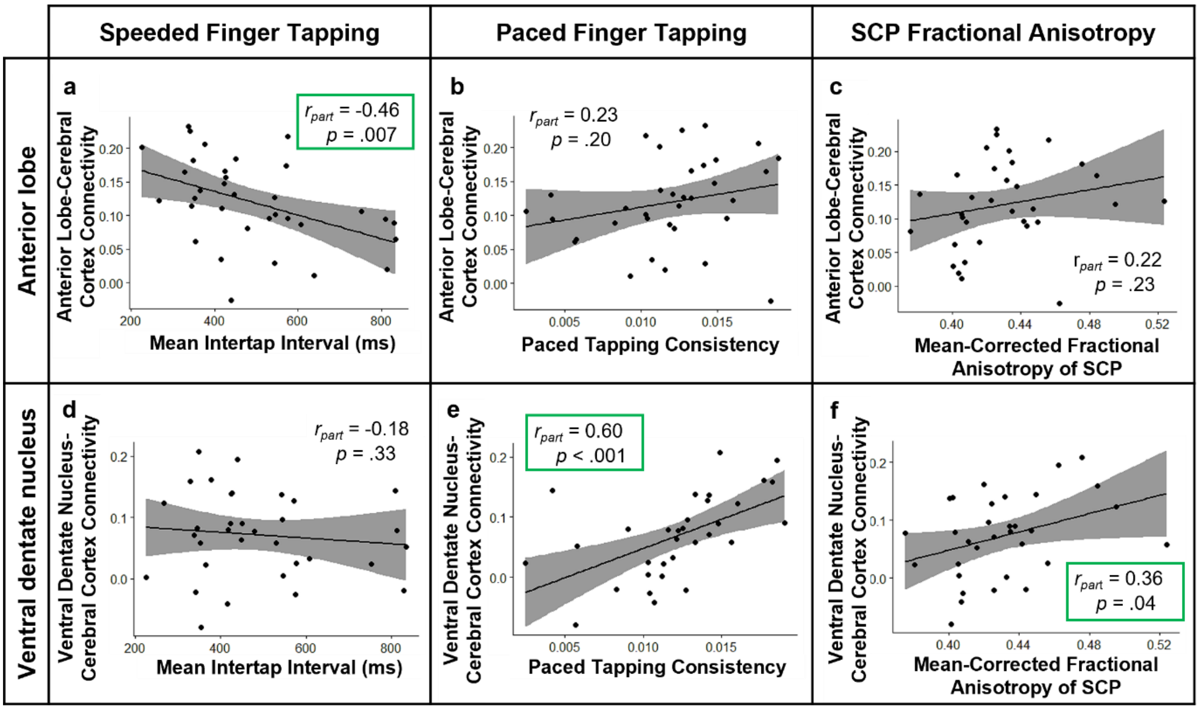

Supplementary Fig. 2 Correlations between cerebello-cerebral connectivity, measures of psychomotor function, and fractional anisotropy of the superior cerebellar peduncles. Statistically significant correlations in this figure are denoted by a green outline. SCP = superior cerebellar peduncles, ms = milliseconds, r_{part} = partial correlation, controlling for the effect of site.

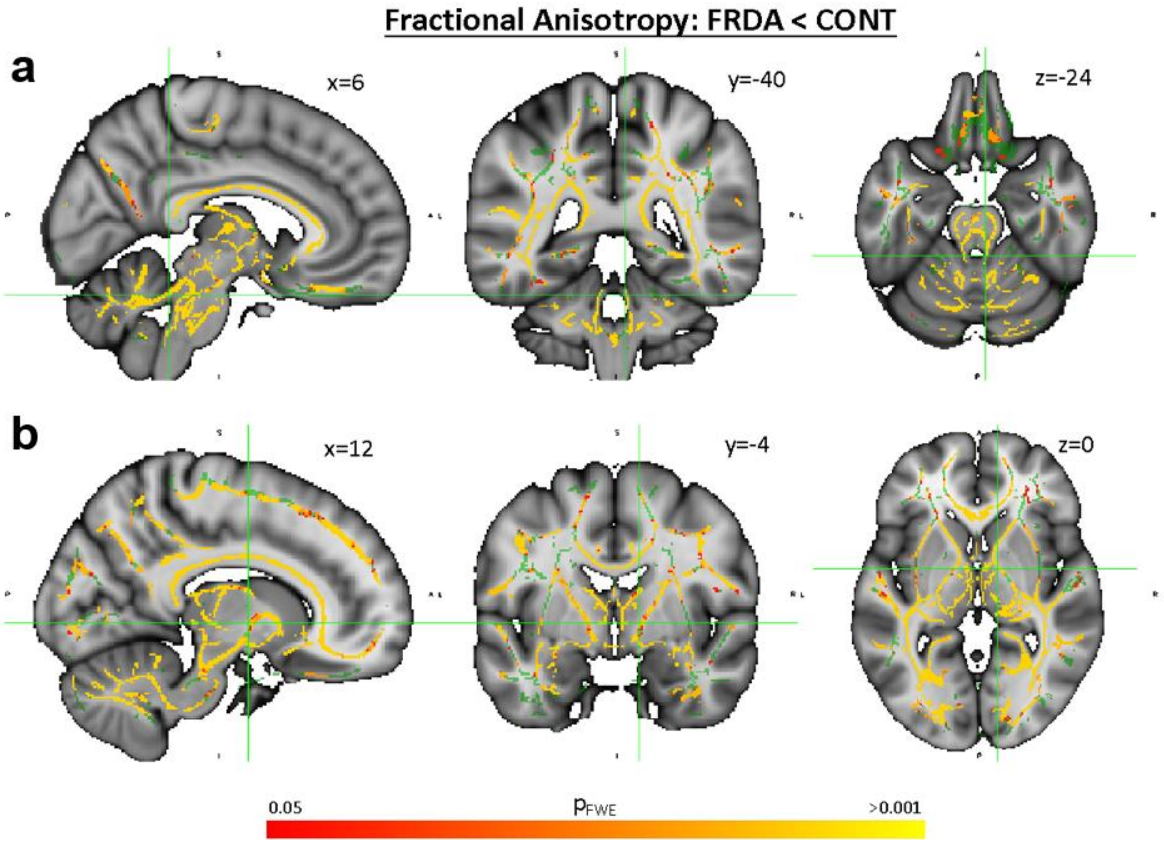

Supplementary Fig. 3 Group differences in fractional anisotropy at the level of the (**a**) cerebellum and (**b**) basal ganglia. Yellow-red colouring represents sections of the white matter skeleton that have significantly smaller fractional anisotropy in people with Friedreich ataxia (FRDA) relative to healthy controls (CONT) at family-wise error-corrected significance ($p_{FWE}<0.05$). Areas of the skeleton that are not significantly different are depicted in green. No areas showed increased fractional anisotropy in the FRDA relative to CONT groups

Supplementary Table 1. FRDA significant between-group cluster values correlated with measures of disease severity, psychomotor function and white matter integrity

	Age at onset		Disease duration		SARA		PTAP		STAP		FA_SCP		FA_MCP		FA_CST	
	r_{part}	p	r_{part}	p	r_{part}	p	r_{part}	p	r_{part}	p	r_{part}	p	r_{part}	p	r_{part}	p
Anterior lobe	-0.04	.84	-0.34	.05	**-0.36**	**.04**	0.17	.35	**-0.41**	**.02**	0.20	.26	0.20	.29	0.29	.10
Superior posterior lobe	0.19	.27	-0.22	.21	**-0.41**	**.02**	0.27	.13	-0.28	.11	0.30	.09	-0.11	.54	0.13	.47

Note. Significant partial correlations (r_{part}) are bolded, with site included as a covariate. SARA = Scale for the Assessment and Rating of Ataxia, PTAP = Paced finger tapping task, STAP = Speeded finger tapping task, FA_SCP = Fractional anisotropy of the superior cerebellar peduncles, FA_MCP = Fractional anisotropy of the middle cerebellar peduncles, FA_CST = Fractional anisotropy of the corticospinal tract.

Supplementary Table 2. Correlating cerebellar connectivity in individuals with FRDA with measures of disease severity, psychomotor function and white matter integrity

	Age at onset		Disease duration		SARA		PTAP		STAP		FA_SCP		FA_MCP		FA_CST	
	r_{part}	p	r_{part}	p	r_{part}	p	r_{part}	p	r_{part}	p	r_{part}	p	r_{part}	p	r_{part}	p
Anterior lobe	**0.02**	**.93**	**-0.35**	**.04**	-0.32	.08	0.23	.20	**-0.46**	**.007**	0.22	.23	0.26	.16	0.31	.09
Superior posterior lobe	0.06	.74	-0.13	.46	-0.21	.25	0.18	.31	-0.19	.30	-0.03	.88	0.04	.82	0.20	.28
Inferior posterior lobe	-0.33	.05	-0.26	.14	-0.09	.61	0.26	.15	0.04	.83	-0.02	.91	0.00	.99	**0.40**	**.02**
Dorsal dentate	0.12	.53	0.24	.18	0.09	.65	0.05	.79	-0.11	.56	-0.25	.18	-0.21	.26	-0.04	.83
Ventral dentate	0.22	.23	0.01	.94	-0.24	.20	**0.60**	**<.001**	-0.18	.33	**0.36**	**.04**	0.21	.25	0.12	.52

Note. Significant partial correlations (r_{part}) are bolded, with site included as a covariate. SARA = Scale for the Assessment and Rating of Ataxia, PTAP = Paced finger tapping task, STAP = Speeded finger tapping task, FA_SCP = Fractional anisotropy of the superior cerebellar peduncles, FA_MCP = Fractional anisotropy of the middle cerebellar peduncles, FA_CST = Fractional anisotropy of the corticospinal tract.

Supplementary Table 3. Correlating healthy control cerebellar connectivity with measures of psychomotor function and white matter integrity

	PTAP		STAP		FA_SCP		FA_MCP		FA_CST	
	r_{part}	p	r_{part}	p	r_{part}	p	r_{part}	p	r_{part}	p
Anterior lobe	0.20	.20	0.02	.89	0.03	.85	-0.00	.99	0.06	.71
Superior posterior lobe	0.14	.38	0.12	.43	0.00	1.00	0.06	.71	0.06	.72
Inferior posterior lobe	0.10	.52	-0.11	.48	0.05	.76	0.17	.26	0.03	.83
Dorsal dentate	0.15	.41	0.17	.36	-0.16	.39	0.05	.77	0.07	.71
Ventral dentate	0.27	.13	-0.06	.75	-0.21	.25	-0.01	.95	-0.09	.61

Note. Site was included as a covariate in the partial correlations (r_{part}). PTAP = Paced finger tapping task, STAP = Speeded finger tapping task, FA_SCP = Fractional anisotropy of the superior cerebellar peduncles, FA_MCP = Fractional anisotropy of the middle cerebellar peduncles, FA_CST = Fractional anisotropy of the corticospinal tract.
